# Supplementary material for: High‐fat diet in a mouse insulin‐resistant model induces widespread rewiring of the phosphotyrosine signaling network
Source: Mol Syst Biol. 2019 Aug 1;15(8):e8849. doi: 10.15252/msb.20198849 (PMC6674232; doi:10.15252/msb.20198849)
Supplement: Supplementary file 1 — Expanded View Figures PDF [file MSB-15-e8849-s001.pdf]

Expanded View Figures

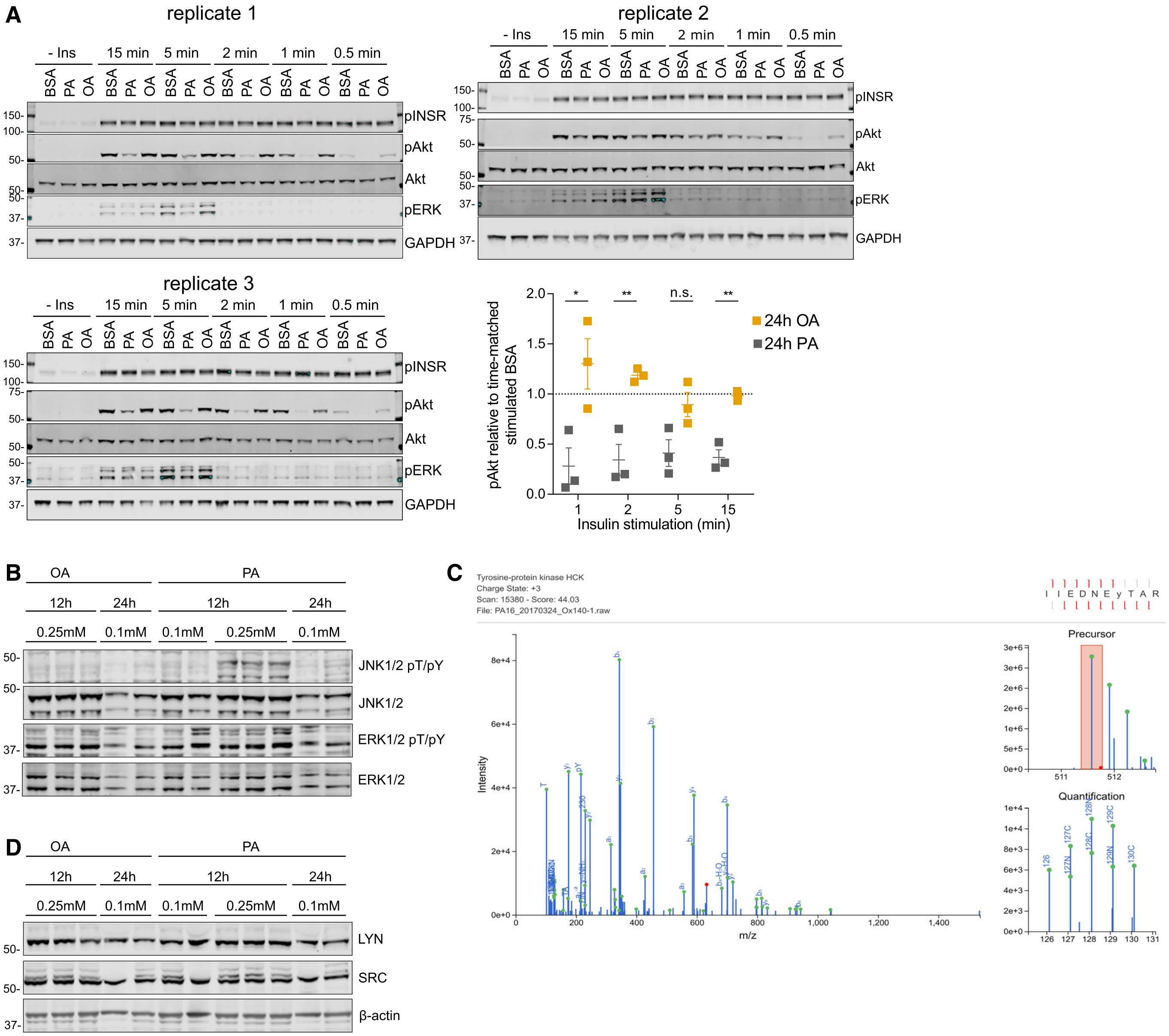

Figure EV1. Effects of FFA on insulin response and MS/MS validation of selected protein and pTyr levels in H4IIE cells.

A All immunoblots corresponding to pAkt levels in presence of OA and PA and loading-controlled ratios relative to time-matched insulin stimulated BSA (mean ± SEM, $n = 3$; *P-value ≤ 0.05; **P-value ≤ 0.01, n.s., not significant using unpaired Student's t-test). Cells were treated with BSA or 0.1 mmol/l BSA-conjugated FFA for 24 h before being stimulated with 100 nmol/l insulin for 0.5–15 min.

B Immunoblot of selected tyrosine sites that were upregulated by PA but not OA in H4IIE cells. Cells were treated with BSA-conjugated PA or OA for indicated times and concentrations before protein extraction. Shown are 2 or 3 independent replicates of each condition. PA, palmitic acid; OA, oleic acid.

C Representative MS/MS spectrum of the common SFK tryptic peptide containing the phosphorylated tyrosine of the activation loop. Precursor-ion, b- and y-ions, TMT-reporter ions, and ions corresponding to peptide fragments with neutral losses were annotated in CAMV and manually validated for every scan of every peptide as detailed in the Materials and Methods section.

D Immunoblot of selected SFK proteins. H4IIE cells were treated with different concentrations of OA or PA for different periods of time as indicated. PA, palmitic acid; OA, oleic acid.

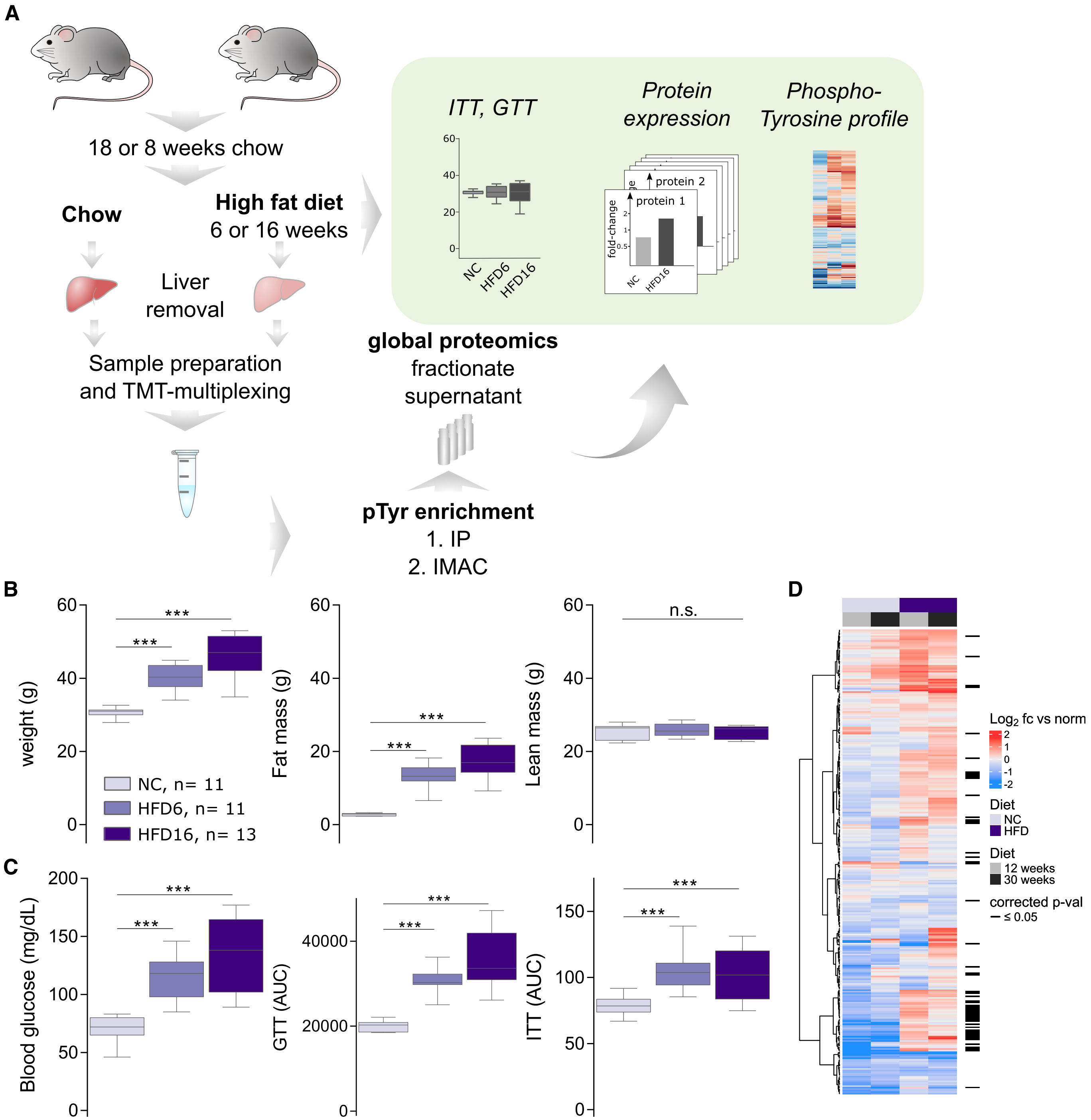

Figure EV2.

 © 2019 The Authors

◀ **Figure EV2. Overview of proteomics study and physiological analysis of NC-fed and HFD-fed mice.**

A Schematic representation of proteomics and phosphoproteomics analysis of HFD and NC livers and phenotypic measurements before sample preparation (data set 1). Male C57BL/6J mice were fed a normal chow (NC) diet for 8 or 18 weeks before switching to a high-fat diet for 16 or 6 weeks, respectively. NC control group, HFD6, and HFD16 were sacrificed at an age of 24 weeks. After indicated diet, mice were starved overnight and phenotypically assessed by measuring body composition, and glucose (GTT) and insulin tolerance (ITT). Mice were then sacrificed to extract the livers which were homogenized, digested to peptides, and tagged by isobaric mass tags for multiplexing. All samples were subjected to a two-step phosphotyrosine enrichment protocol. The IP flow-through of HFD16 and NC samples (3 each) was fractionated by high-pH reversed-phase chromatography, and phosphotyrosine and fractionated samples were analyzed by nano-spray LC-MS/MS.

B The body mass of NC-fed and 6- and 16-week HFD-fed mice was measured. Fat and lean mass were measured by ^{1}H-MRS analysis (median + quartile3 + 1.5*IQR/median – quartile1-1.5*IQR; *n* = 11–13; ***$P < 1e{-}3$; n.s., not significant, using unpaired Student's *t*-test, two-sided).

C NC-fed and 6- and 16-week HFD-fed mice were fasted overnight and the blood concentration of glucose was measured (median + quartile3 + 1.5*IQR/median – quartile1-1.5*IQR; *n* = 11–13; ***P-value $< 1e{-}03$). Insulin tolerance (ITT) and glucose tolerance (GTT) tests were performed using NC-fed mice and 6- and 16-week HFD-fed mice (mean ± SEM; *n* = 11–13; ***P-value $< 1e{-}03$, using unpaired Student's *t*-test, two-sided).

D Unsupervised hierarchical clustering of study 2 pTyr peptide ratios (log_2) relative to a normalization sample. Mice were kept on a HFD alongside control animals on standard chow (*n* = 2 per condition). Livers were frozen immediately after animals had been sacrificed, and pTyr analysis was run as described above. Statistical significance of difference between corresponding NC and HFD was assessed using a combination of standard and moderated *t*-test and rank products (P-value < 0.05 after Benjamini-Hochberg adjustment).

© 2019 The Authors

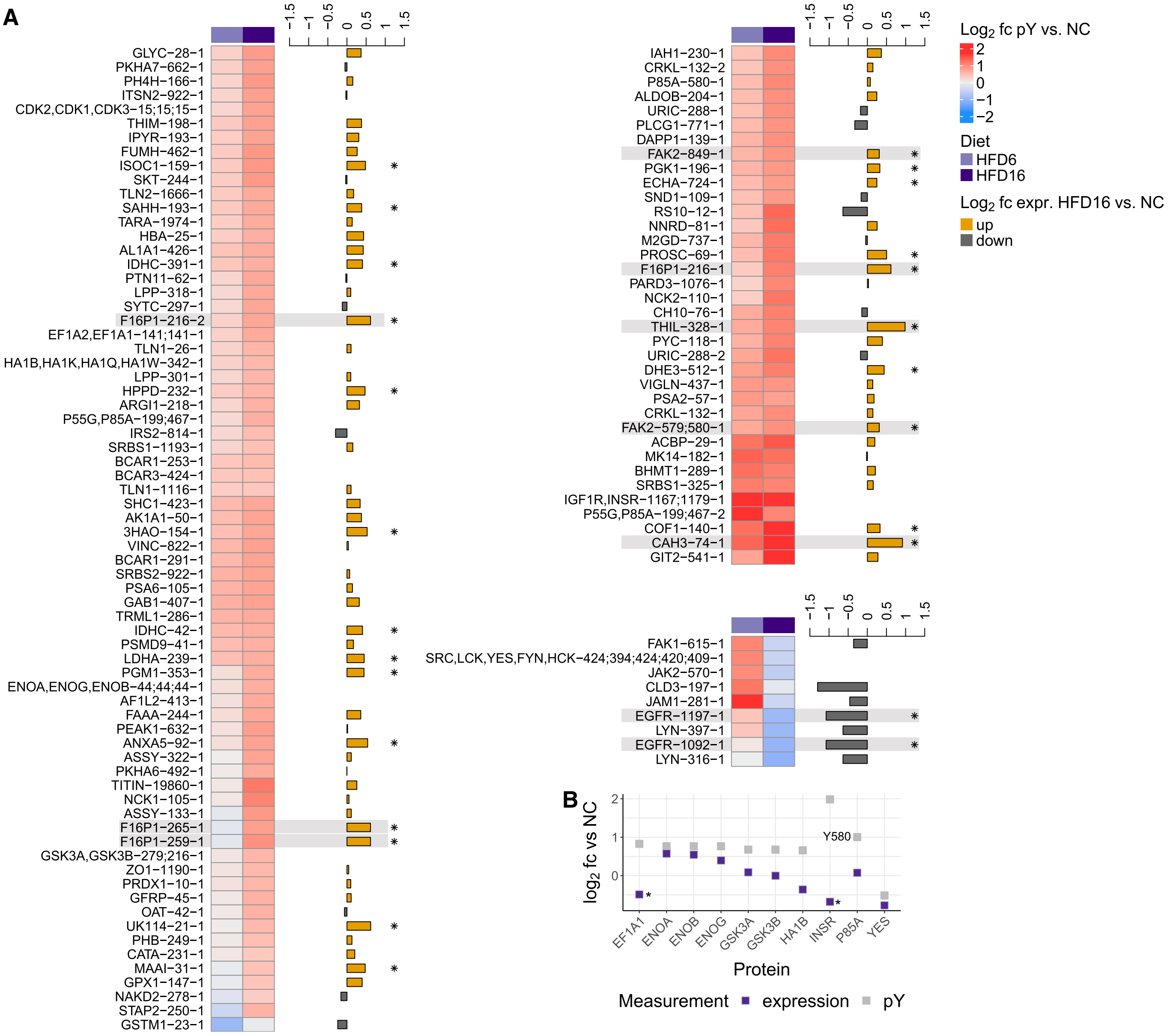

Figure EV3. Effects of diet on protein expression and tyrosine phosphorylation.

A Data set 1 average ratios ($\log_2$) relative to NC of pTyr sites significantly different in any pairwise comparison (Fig 2A, *P*-value < 0.05 after Benjamini-Hochberg adjustment, n = 2–4), and corresponding average protein abundances in HFD16 relative to NC ($\log_2$, n = 2–3). Clusters are generated using hierarchical clustering of significantly different phosphopeptide ratios. Protein abundances were determined by LC-MS/MS of fractionated pTyr IP supernatant. Highlighted sites are mentioned in the text (* - protein expression *P*-value < 0.05).

B Data set 1 expression and pTyr level in HFD16 relative to NC ($\log_2$) of proteins whose expression could not be displayed in Fig EV3A due to non-unique peptides found in multiple proteins (* - protein expression *P*-value < 0.05).

 © 2019 The Authors

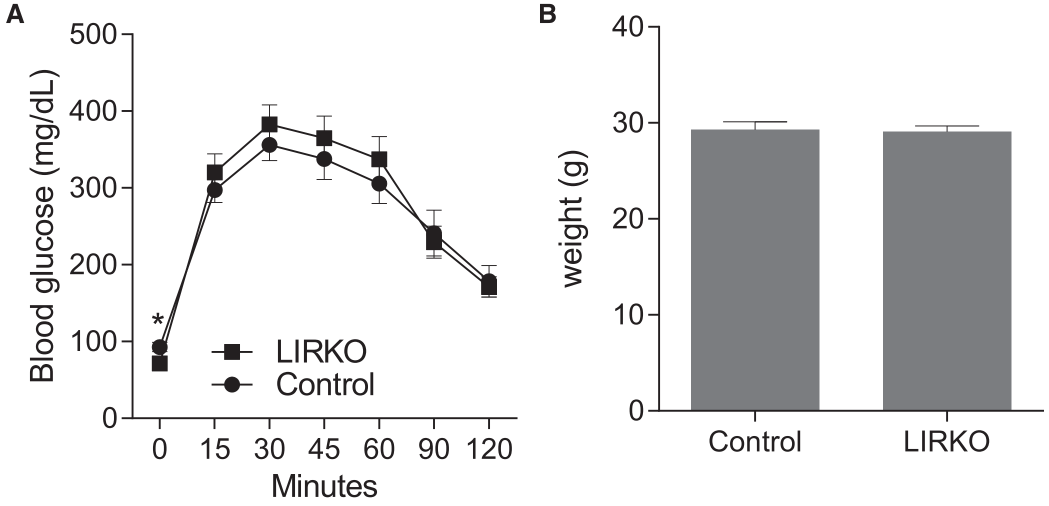

Figure EV4. Phenotypic parameters of control and LIRKO mice.

A, B NC-fed control and LIRKO mice were examined at age 24 weeks using a glucose tolerance test (GTT, A) and weight measurement (B) (mean ± SEM; n = 9–15, * P-value < 0.05, unpaired Student's t-test, two-sided).

Figure EV5. Effects of antioxidants and dasatinib on pTyr levels, insulin response, and phenotypic parameters *in vitro* and *in vivo*.

A, B Unsupervised hierarchical clustering of pTyr peptide levels from cells treated with 0.1 mmol/l OA or PA for 12 h alone or in the presence of antioxidants (A) or increasing concentrations of dasatinib (B). Abundances are expressed as ratios (log_2) of treatment relative to BSA. BHA—200 µmol/l; Rotenone—10 nmol/l; NAC—5 mmol/l.

C–E Immunoblots of Akt phosphorylation (S473) after insulin stimulation in the presence of BSA or FFAs with or without increasing concentrations of NAC (C), dasatinib (D), or BHA (E). Cells were incubated with BSA or 0.1 mmol/l FFA media and inhibitors for 12 h and then stimulated with 100 nmol/l insulin for 5 min. Lanes marked with * indicate samples that were run on same blot in (C) and (D). Ins, insulin

F Quantification summary for pAkt (S473) of NAC, BHA, and dasatinib-treated cells. H4IIE cells were treated with BSA or 0.1 mmol/l FFA for 12 h in the presence of increasing concentrations of NAC, BHA, or dasatinib before being stimulated with 100 nmol/l insulin for 5 min. Loading-controlled pAkt levels were expressed relative to insulin stimulated BSA (mean ± SEM; n = 3; **P-value < 0.01; n.s., not significant using unpaired Student's t-test). PA, palmitic acid; OA, oleic acid.

G Reactive oxygen (ROS) levels after treatment with 0.1 mmol/l FFA for 8 h in the presence or absence (FFA only) of dasatinib. Cells were grown in the presence of FFA or BSA alone for indicated times and co-treated with different concentrations of dasatinib. ROS levels were measured and expressed as ratios relative to time-matched BSA (mean ± SEM; $n \geq 3$; *P-value < 0.05; **P-value < 0.01; n.s., not significant using unpaired Student's t-test, two-sided). PA, palmitic acid; OA, oleic acid.

© 2019 The Authors

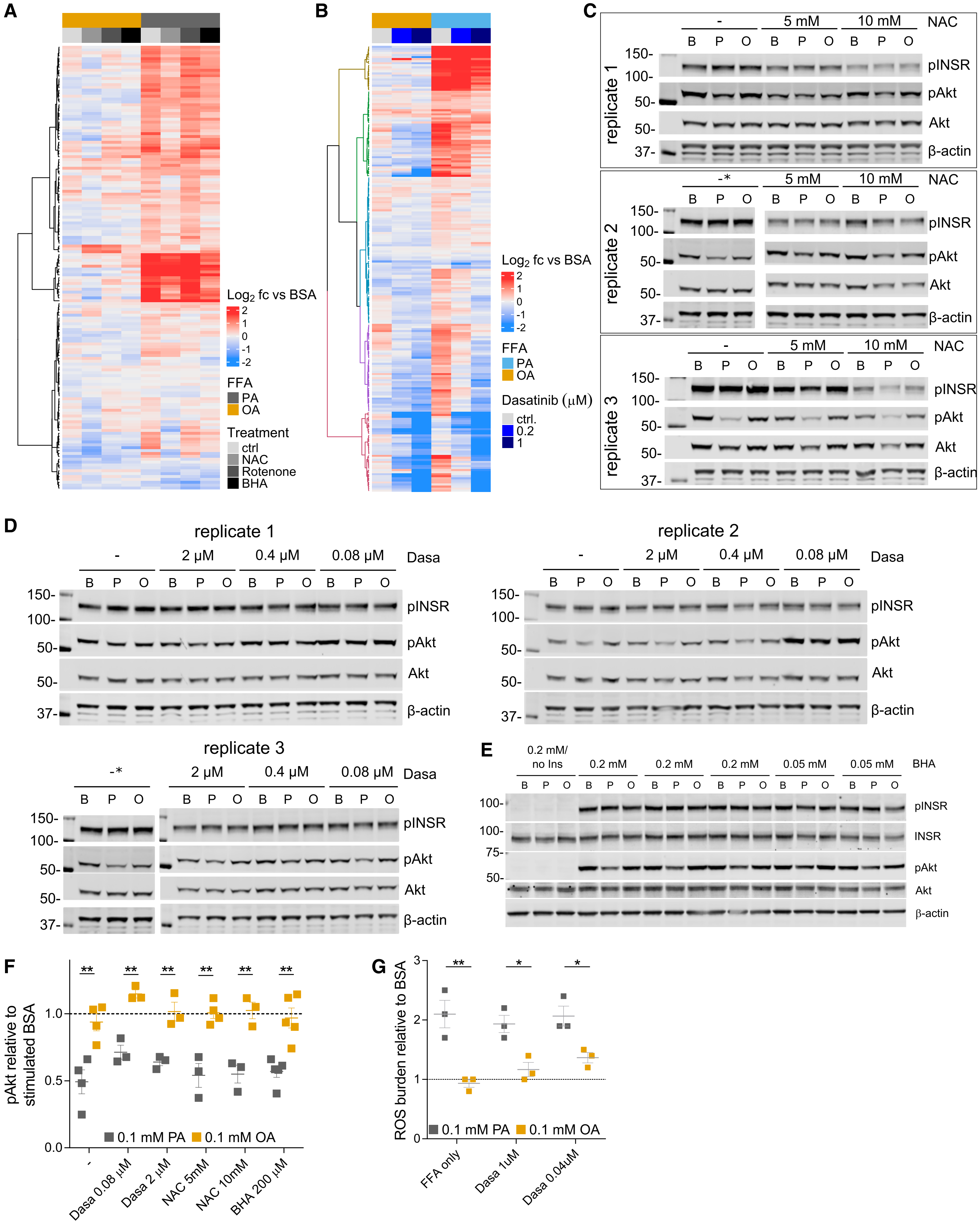

Figure EV5.

© 2019 The Authors

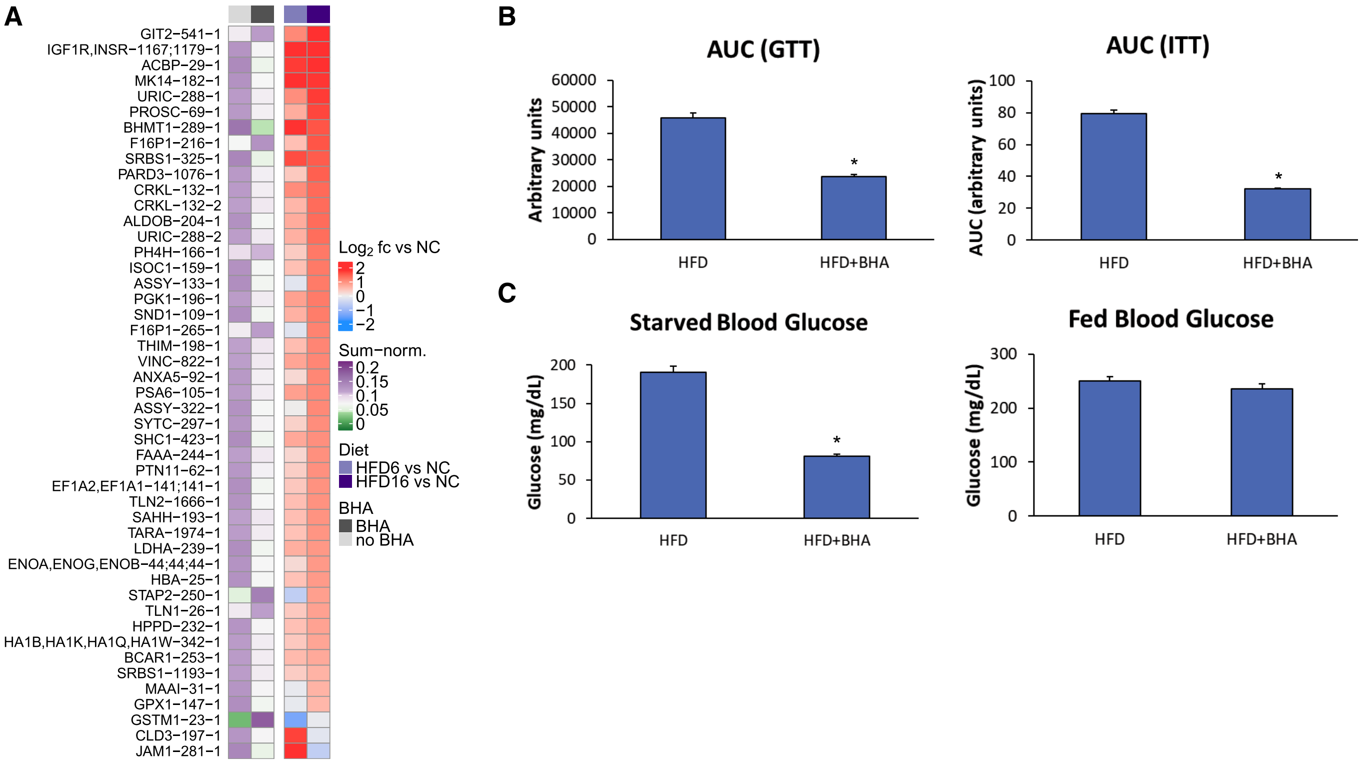

Figure EV6. Effects of antioxidants and dasatinib on pTyr levels, insulin response, and phenotypic parameters *in vitro* and *in vivo*.

A Heatmap of pTyr peptides significantly affected by BHA (left panel) and by HFD (right panel). Number after gene name corresponds to tyrosine position in mouse sequence, while the last number corresponds to different peptide form (e.g., methionine oxidation, missed cleavage site).

B AUC of glucose tolerance test (GTT) and insulin tolerance test (ITT) of HFD-fed mice with or without BHA supplementation to the diet ($n = 10$; mean ± SEM; *P-value < 1e-03 using unpaired Student's t-test, two-sided).

C Fasted and fed blood glucose levels in HFD-fed mice with or without BHA supplementation to the diet ($n = 10$, mean ± SEM; *P < 1e-03 using unpaired Student's t-test, two-sided).

© 2019 The Authors
